# Evidence for ubiquitous preferential particle orientation in representative oceanic shear flows

**DOI:** 10.1002/lno.10618

**Published:** 2017-07-18

**Authors:** Aditya R. Nayak, Malcolm N. McFarland, James M. Sullivan, Michael S. Twardowski

**Affiliations:** ^1^ Harbor Branch Oceanographic Institute at Florida Atlantic University Fort Pierce Florida

## Abstract

In situ measurements were undertaken to characterize particle fields in undisturbed oceanic environments. Simultaneous, co‐located depth profiles of particle fields and flow characteristics were recorded using a submersible holographic imaging system and an acoustic Doppler velocimeter, under different flow conditions and varying particle concentration loads, typical of those found in coastal oceans and lakes. Nearly one million particles with major axis lengths ranging from ∼14 μm to 11.6 mm, representing diverse shapes, sizes, and aspect ratios were characterized as part of this study. The particle field consisted of marine snow, detrital matter, and phytoplankton, including colonial diatoms, which sometimes formed “thin layers” of high particle abundance. Clear evidence of preferential alignment of particles was seen at all sampling stations, where the orientation probability density function (PDF) peaked at near horizontal angles and coincided with regions of low velocity shear and weak turbulent dissipation rates. Furthermore, PDF values increased with increasing particle aspect ratios, in excellent agreement with models of spheroidal particle motion in simple shear flows. To the best of our knowledge, although preferential particle orientation in the ocean has been reported in two prior cases, our findings represent the first comprehensive field study examining this phenomenon. Evidence of nonrandom particle alignment in aquatic systems has significant consequences to aquatic optics theory and remote sensing, where perfectly random particle orientation and thus isotropic symmetry in optical parameters is assumed. Ecologically, chain‐forming phytoplankton may have evolved to form large aspect ratio chains as a strategy to optimize light harvesting.

Oceanic particles important for biogeochemical and optical studies consist of a variety of inorganic material, organic detritus, and living organisms, that encompass a diverse range of shapes and vary in size from sub‐microns (e.g., picoplankton) to a few cm (e.g., colonial diatom chains) (Lal 1977). Due to their ubiquitous presence, they influence areas of interest spanning aspects of ocean sciences as diverse as sediment transport, marine ecology, climate change, remote sensing, and ocean optics. For example, quantification of sediment resuspension and transport by waves and currents in coastal bottom boundary layer environments are critical to coastal engineering applications (Grant and Madsen 1986; Fredsøe and Deigaard 1992; Hsu et al. 2007). A key aspect of climate change studies is understanding how oceans act as sinks for anthropogenic carbon, where the absorbed organic carbon is transported by sinking particulate matter from the upper ocean to the seabed through a process known as the “biological pump” (Honjo et al. 2014; Turner 2015).

The inherent optical properties (IOPs), which control the propagation of light in water are strongly influenced by the composition of local particulate matter (Bohren and Huffman 1983; Jonasz and Fournier 2007). Both active and passive remote sensing rely on interpreting the signature of backscattered light from the ocean to detect algal biomass, “thin layers” with high phytoplankton concentration, suspended particle mass, and to quantify biological primary productivity (Platt and Satyendranath 1988; Stramski and Kiefer 1991; Twardowski et al. 2007; Churnside et al. 2014). Thus, characterizing oceanic particulates based on type, size, and shape has been the focus of a multitude of studies over the past several decades (Jackson et al. 1997; Boss et al. 2001; Twardowski et al. 2001; Groundwater et al. 2012; Twardowski et al. 2012).

However, a specific aspect of particle‐flow interaction, i.e., whether particles exhibit preferential orientation when exposed to typical oceanic shear flows, remains relatively unexplored, despite having significant consequences to numerous bio‐optical and ecological studies. For example, using a coupled fluid‐optical model and laboratory experiments, Marcos et al. (2011) showed that preferential alignment of microorganisms in response to shear could increase optical backscattering by 20–30%, depending on the shear strain rate magnitudes. From an ecological perspective, if chain‐forming phytoplankton with large aspect ratios orient themselves to a flow direction perpendicular to the downwelling light field, i.e., expose more surface area to the sunlight, primary productivity could be increased (Bricaud and Morel 1986). Nonrandom particle orientation in the atmosphere and the resulting optical effect (e.g., oriented atmospheric ice crystals generating a “sun dog” halo) is a well‐known phenomenon (Takano and Liou 1990; Noel and Chepfer 2004). However, the underlying assumption in ocean optics theory (e.g., radiative transfer models) is that oceanic particles show ideal random orientation. For instance, in virtually all models and measurements, to the best of our knowledge, the volume scattering function is assumed to have azimuthal symmetry, i.e., no dependency on azimuthal viewing direction, which inherently implies that the particle population is randomly oriented. Increasingly, this critical assumption has been challenged by a handful of recent modeling and experimental studies on particle orientation in shear flows (Karp‐Boss and Jumars 1998; Marcos et al. 2011; Talapatra et al. 2013).

The mathematical description of the orbital motion of spheroidal particles in a laminar flow with constant shear, wherein particles spend longer durations oriented along the flow direction was formulated by Jeffery (1922). Several numerical and experimental studies on particle suspensions in shear flows are in agreement with Jeffery's theory (Hinch and Leal 1972; Ingber and Mondy 1994). However, only fairly recently has Jeffery's theory been considered in the context of marine particles. Laboratory studies on both motile and nonmotile phytoplankton have shown that particles exhibit preferential orientation under typical oceanic shear conditions (Karp‐Boss and Jumars 1998; Karp‐Boss et al. 2000; Marcos et al. 2011; Barry et al. 2015). However, to the best of our knowledge, nonrandom orientation has been reported in only two prior field measurements studying particles in natural, undisturbed oceanic waters (Malkiel et al. 1999; Talapatra et al. 2013). In both studies, particle orientation was not the primary focus and sample sizes were much smaller, precluding a detailed analysis of this phenomenon. Thus, a comprehensive analysis of particle orientation under varying mean shear/stratification and turbulence conditions throughout the water column is still lacking.

Paucity of data could be attributed to significant challenges in studying marine particles in situ in undisturbed flow environments. The goal of the current study was to bridge this gap in data by using a combination of simultaneous flow measurements, particle characterization and bio‐optical profiles, while ensuring that the natural environment remained as undisturbed as possible. Holography is currently the only viable nonintrusive technique that can fully characterize oceanic particle distributions over a three‐dimensional (3D) sample volume (Hobson and Watson 2002; Pfitsch et al. 2005). Briefly, this technique involves illuminating a sample volume with a coherent and collimated beam of light, and recording the diffraction patterns resulting from interference between light scattered by particles in the volume and the undisturbed light beam (Vikram 1992). Subsequent numerical reconstruction using the hologram diffraction patterns at different planes/sections within the sample volume provides in focus images of all particles within the volume, thus providing 3D information on the shape, spatial distributions, and motions of particles and organisms. A comprehensive overview on digital holography and applications can be found in Schnars and Jueptner (2005) and Katz and Sheng (2010). Several configurations of film‐based (Katz et al. 1999; Malkiel et al. 1999) and subsequently, digital holographic systems (Owen and Zozulya 2000; Pfitsch et al. 2005; Bochdansky et al. 2013; Talapatra et al. 2013) have been employed in the ocean over the last decade and a half, with the in‐line holographic setup used in our experiments representing the latest generation system.

Herein, field experiments are described involving a comprehensive dataset collected over a wide range of environmental conditions and particle densities, using a state‐of‐the‐art in‐line digital holographic system and other optical and acoustic sensors. In the “Field measurements” section, we describe the field measurements, instrumentation suite, and the flow/environmental conditions. In the “Data analysis methods” section, results from analysis of several datasets, including particle size distributions (PSDs), particle orientation and associated correlations with small scale shear and turbulence structure, spanning the entire water column depth, are presented and compared to prior theoretical/laboratory studies. We conclude by presenting a brief summary of the work in the “Results and discussion” section.

## Field measurements

### Instrumentation suite

**Figure 1:**
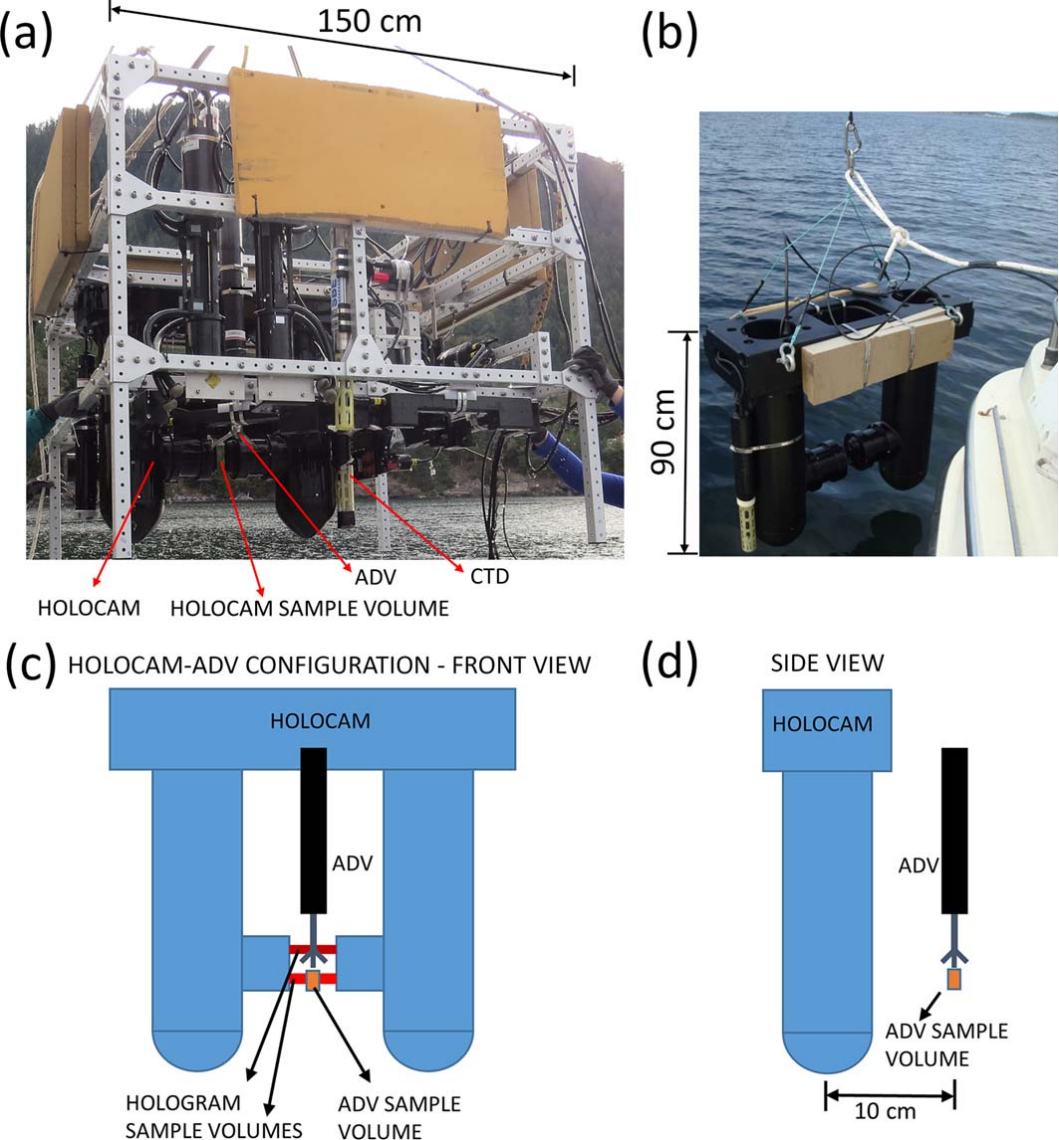
(**a**) The instrumentation package being deployed at East Sound, Washington in September 2015; (**b**) a close‐up view of the HOLOCAM; (**c**) Front view of relative placement of the HOLOCAM and ADV; and (**d**) Side view of HOLOCAM‐ADV placement.

A suite of optical and acoustic instrumentation was assembled to concurrently measure the IOPs, particle characteristics (e.g., size distribution, aspect ratio, orientation), and local small‐scale flow structure. The instruments, which were mounted on an approximately 1.5 m × 1.5 m square frame, ∼1.2 m in height (Fig. 1a), are described briefly below. This profiling system was made slightly negatively buoyant relative to seawater, by adding flotation foam on all sides of the package, as seen in Fig. 1a. The system was deployed using the “slow‐drop” technique (Cowles et al. 1998; Sullivan et al. 2010), and slowly hand lowered on a cable, at a descent rate of 5–6 cm s^−1^. This method helped achieve a free, consistent descent rate, independent of a ship's normal winch controlled hydro‐wire, and associated ship rolls/motions. It should also be noted that the ship was anchored during data acquisition at all stations, thus any drift‐associated influences to the measurements were minimal. The submersible holographic imaging system (HOLOCAM) enabled quantification of 3D particle fields in situ in undisturbed flow (Fig. 1b). The mechanical design incorporates several elements to minimize flow disturbances. The two instrumentation towers, 20 cm in diameter, are connected to streamlined fins, with the sample volume lying between the bulbous fin tips, which were designed to reduce flow separation when the relative flow is inclined. Vortex rollup in the gap is also avoided by pushing it to the aft side when the relative flow to the fin is at an angle. The Reynolds number ( $Re=wD_{H}/\nu$, where *w* and $D_{H}$ are the velocity and length scales, and ν is the kinematic viscosity of seawater, taken as 1.1 × 10^−6^ m^2^ s^−1^) for this body in motion, using the diameter of the tower ( $D_{H}$ = 20 cm) and the rate of descent (*w* = 5 cm s^−1^) as the relevant length and velocity scales respectively is ∼9000. The optical configuration consisted of two in‐line holography setups simultaneously studying sample areas of different magnifications in close proximity. A 660 nm Nd‐YAG laser was used as the illumination source, wherein the laser beam was spatially filtered and collimated before being passed through a beamsplitter. The resulting twin beams were then directed through separate sampling windows, illuminating the 4 cm long sample length over the two regions of interest. The laser with this particular wavelength was chosen as red light attenuates fast and most marine organisms, including motile plankton, are least sensitive to red light (Forward 1988; Buskey et al. 1989). Furthermore, only weak side scatter could be observed by an organism not in the sample volume, which is typically overwhelmed by ambient light in the euphotic zone. The low magnification data was recorded on an Imperx 2048 × 2048 pixel CCD camera, imaging a field of view (FOV) of 9.4 × 9.4 mm, corresponding to a resolution of 4.59 μm per pixel. The high magnification holograms were imaged using a JAI 2432 × 2058 pixel camera, with the FOV spanning 0.83 × 0.7 mm, corresponding to a resolution of 0.34 μm per pixel. Thus, the sample volumes imaged correspond to 3.53 mL and 23.2 μL, respectively, for each low and high magnification hologram. This dual‐magnification design could be used to resolve particle sizes over nearly 4 orders of magnitude. Only low magnification data were considered in this study. The images, acquired at a rate of 15 Hz, were transmitted through optical fiber to the on‐deck computer, where they were viewed in real‐time while simultaneously being stored in an array of hard drives. A Sea‐Bird Electronics 49 Fastcat conductivity temperature depth sensor (CTD) mounted alongside the HOLOCAM, provided simultaneous records of salinity, temperature, density, and depth during each cast. In order to correlate the small‐scale flow physics to the observed particle orientation, it is imperative to have concurrent, co‐located velocity measurements within the measured particle fields. An acoustic Doppler velocimeter (ADV, Nortek Vector) was mounted on the instrument frame, such that its sample volume was parallel to and at the same depth as the center of the HOLOCAM sample volume (Fig. 1c,d), though offset in the lateral direction by 10 cm. The pressure sensor on the ADV also provided a second, independent record of system depth. Both the HOLOCAM and ADV were mounted facing downwards, such that their sample volumes were able to measure undisturbed particle/flow fields.

Optical scattering measurements were made using a host of sensors with sample volumes all positioned at the same depth in different orientations. Only a WET Labs ac‐9 multi‐spectral absorption and attenuation meter is relevant to this study, with calibration and processing protocols described in Twardowski et al. (1999). Data from all sensors along with a Sea‐Bird Electronics 49 Fastcat CTD were recorded simultaneously, timestamped and archived on WET Labs DH‐4 data loggers for future data extraction, merging, and processing.

### Field site and environmental conditions

Field experiments were performed in East Sound, WA, a fjord with partial sill in the San Juan Islands in the US Pacific Northwest. The study area is well characterized, with several previous collaborative, large scale field efforts to study biophysical interactions and optical and acoustic properties (Alldredge et al. 2002; McManus et al. 2003; Menden‐Deuer 2008; Talapatra et al. 2013). Over the span of nearly 2 weeks in September 2015, the instrument package recorded data at 50 stations at varying locations across East Sound. For most stations, both a downcast and upcast were recorded; downcasts only were collected for four stations. Pertinent details for the four datasets chosen for analysis in this manuscript are as follows: Sta. 39 and 40 were recorded between 09:04–09:15 a.m. and 12:15–12:32 p.m. (all times in local Pacific Daylight Time) on 21 September 2015 at 48.61°N, 122.85°W, while Sta. 46 and 48 were recorded between 08:59–09:12 a.m. and 11:14–11:23 a.m. on the following day at 48.64°N, 122.85°W, approximately 2.2 km away. Based on NOAA tidal data, Rosario, Eastsound (Station ID # 9449771), experiences a mixed semi‐diurnal tide, where the peak tidal height is ∼2.1 m above Mean Low Lower Water. On 21 September, the low and high tides between which Sta. 39 and 40 were recorded occurred at 04:45 a.m. and 01:23 p.m., whereas on 22 September, corresponding low and high tides occurred at 05:41 a.m. and 02:15 p.m., respectively, with Sta. 46 and 48 recorded between. The mean depths during these measurements were 23 m at Sta. 39 and 40, and 28 m at Sta. 46 and 48. More details regarding the physical conditions and forcing mechanisms at Eastsound are provided in Dekshenieks et al. (2001) and McManus et al. (2003). After “slow drop” downcasts, upcasts were relatively fast (> 12 cm s^−1^), with flows completely well‐mixed due to the sensor being in the wake of the instrument package. Each profile typically lasted 7–15 min, corresponding to 6300–13,500 holograms per camera. Over the entire deployment, roughly half a million holograms (including both magnifications) were collected, creating a database ∼2.5 TB in size. This wealth of data could be used to explore a wide range of scientific questions related to biophysical interactions, phytoplankton ecology, predator/prey interactions, and ocean optics.

## Data analysis methods

Applied holographic data processing routines can be roughly divided into three steps: background subtraction, reconstruction, and image plane consolidation. First, using all the holograms at a given station, the ensemble averaged image was computed and subtracted from each individual hologram over the entire sample. This enabled us to effectively remove nonuniformities in the images associated with variations in background intensity. Each resultant filtered image was then subjected to numerical reconstruction using the Fresnel diffraction formula (Katz and Sheng 2010), which yielded in‐focus images at different planes over the entire 4 cm length of the sample volume. Reconstruction was done in incremental steps of 500 μm, resulting in 70 planes per hologram (the 10 planes within 2.5 mm of either sampling window were discarded, to avoid any possibility of window boundary layer effects). In‐focus particles within reconstructed planes consist of low intensity pixels with sharp boundaries, while out‐of‐focus particles have much higher pixel intensities and diffuse boundaries. The final step involved generating a composite image where the in‐focus particles at different reconstructed planes were consolidated. The simplest way to do this is to choose the minimum intensity of each pixel over all the reconstructed planes. However, a more effective approach was as follows. Each reconstructed 2048 × 2048 plane was sub‐divided into smaller 128 × 128 sub‐regions. For each sub‐region, the plane with the maximum number of pixels below a certain threshold corresponded to the location of the in‐focus particle. The same criterion was applied to all the 256 windows, collapsing all the in‐focus sub‐regions into one single composite image for further analysis. For large particles traversing window boundaries, this allowed the optimal plane of focus to change along a particle's extent, maintaining sharp particle edges in the composite image. Windows were kept small enough to ensure portions of large particles within window boundaries were entirely in focus and slight discontinuities at window boundaries had minimal impact on segmentation. These steps were then repeated for each hologram in the dataset. Once each composite image was generated, a 2 × 2 low pass frequency filter was applied to reduce noise prior to segmentation. Only objects at least 5 pixels in size (corresponding to area greater than 105 μm^2^) were considered as “particles,” to improve the reliability of the statistics. Below this range, the distinction between noise and actual particles was not always clear. Binary thresholding and subsequent image segmentation analysis was used to generate particle lists with spatial location, area, equivalent diameter, length, aspect ratio, convex hull, and orientation with respect to the horizontal axis of the image plane. Overlapping particles within composite images pose a potential problem for this analysis since they can be counted and analyzed as a single particle, and were separated as follows. The individual depth planes from the reconstruction process were stored for post‐analysis. This provided the intensity variation in a particular pixel with depth. One can proceed with the usual post‐processing steps including forming composite images for segmentation and particle analysis, and then calculate the ratio of the convex hull area to the pixel‐based area for each particle. For overlapping particles, this ratio will be quite high, thus enabling their isolation from all the images. In cases where particle overlap was found to exist, closer examination of the intensity gradient (for each pixel) in the depth direction was carried out in the vicinity of the overlap. Two intensity peaks were observed, corresponding to the in‐focus depths of the particles, which could then be isolated accordingly. If a combination of these methods was not successful in getting two independent particles, both particles were eliminated from the analysis. In general, < 0.3% of the total particles overlapped, and the majority occurred within the “thin layer,” where particles were most abundant.

**Figure 2:**
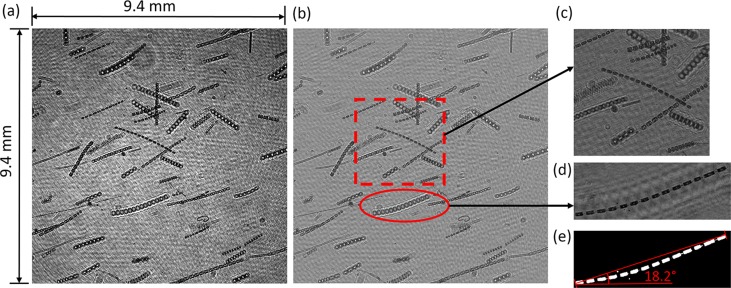
Panel illustrating the hologram reconstruction technique. (**a**) A raw sample hologram; (**b**) the same hologram after background subtraction to remove nonuniformities in image; (**c**) a zoomed in version of the highlighted area reconstructed at *z* = 0 mm (focal plane); (**d**) reconstructed in‐focus particle at *z* = 18 mm; and (**e**) same particle in highlighted area in (**d**) after image segmentation, with associated major axis length and orientation.

An example of the hologram processing routine is shown in Fig. 2, with the raw (Fig. 2a) and background subtracted (Fig. 2b) holograms, respectively. Figure 2c,d show the in‐focus particles at two different reconstruction depths in the sample volume, and Fig. 2e shows an example of the same particle in Fig. 2d, undergoing the segmentation process followed by computation of the particle statistics, including orientation and length. It can be seen that the individual diatom cells in the chain, were stored as discrete particles in the segmented image (due to the narrow cell linkages being above the intensity threshold). However, by calculating the distance between all discrete particles in the neighborhood and re‐connecting ones within a certain distance of each other, the chain was treated as one particle for purposes of calculating particle statistics. Note the length of slightly curved diatom chains (as in Fig. 2e) tends to be underestimated due to fitting a straight line to particle extrema, which is essential to calculate a major axis, minor axis, and corresponding aspect ratio.

ADV and CTD data, both acquired at 16 Hz, were resampled at 15 Hz (HOLOCAM sampling frequency) and time‐stamp matched with the holograms to ensure accurate data synchronization. The customized ADV has an in‐built inertial motion unit (IMU), which enabled us to filter out the system motions from the recorded velocity (e.g., Thomson et al. 2015). The motion correction routines applied were provided by the open source Dolfyn package. It should be noted that the IMU data, while effective in removing high frequency motion, cannot completely separate the low frequency motion (< 0.03 Hz) effectively, due to the bias‐drift inherent to IMU accelerometer measurements.

## Results and discussion

The four stations which are analyzed were chosen to represent different flow and stratification conditions as well as varying particle abundances in the water column. The flow structure and turbulence, as observed in vertical profiles of mean shear and turbulent kinetic energy (TKE) dissipation rates, also differed substantially between the different days. Particle statistics, including PSDs, orientation as a function of depth and aspect ratios, and associated correlations to the simultaneously quantified mean velocity shear and turbulence structure are discussed in detail.

### Stratification and chlorophyll *a* profiles

**Figure 3:**
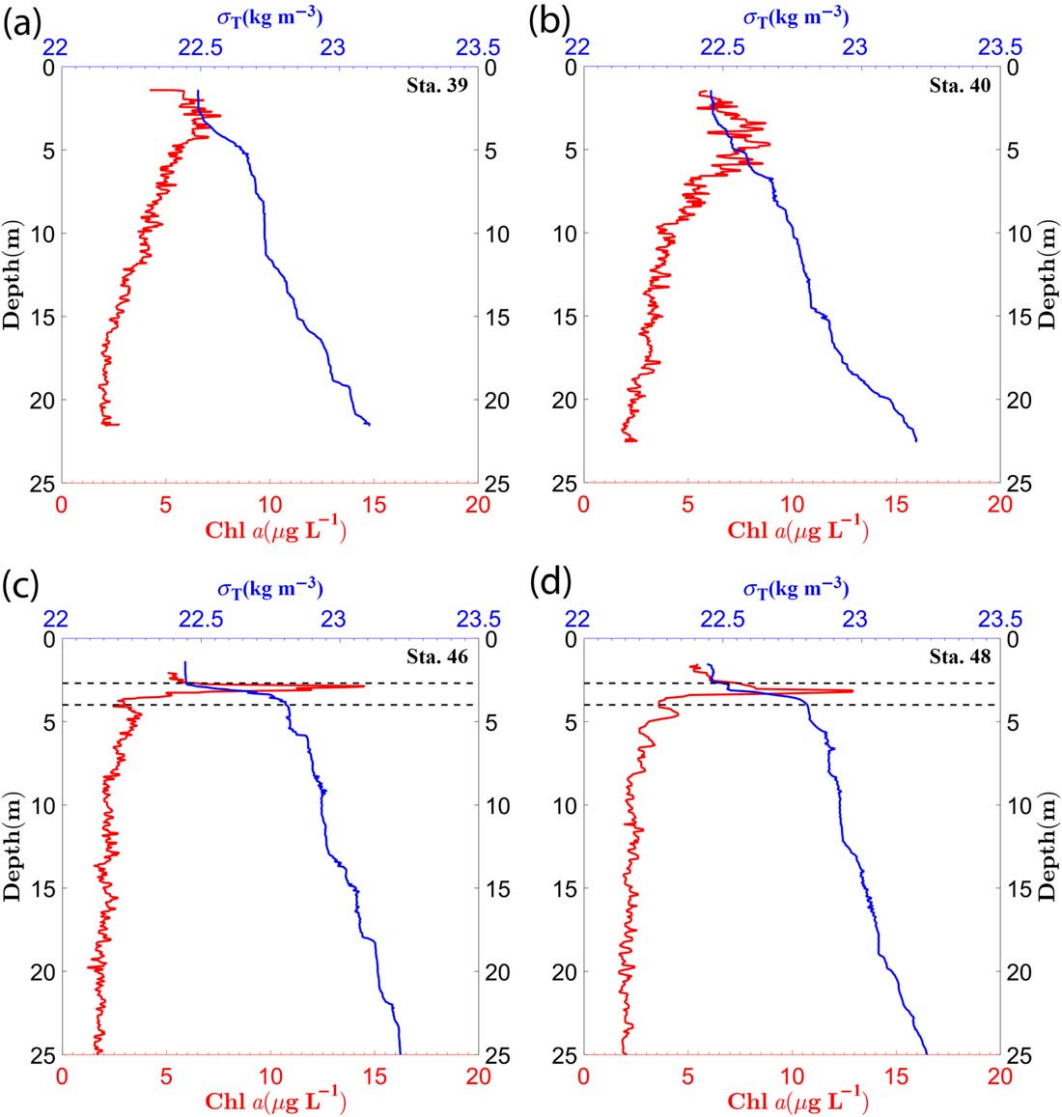
Chl *a* (red) and $\sigma_{T}$ (blue) profiles for (**a**) Sta. 39; (**b**) Sta. 40; (**c**) Sta. 46; and (**d**) Sta. 48. Dashed lines at 2.7 m and 4 m for Sta. 46 and 48 indicate the range of the “thin layer.”

**Figure 4:**
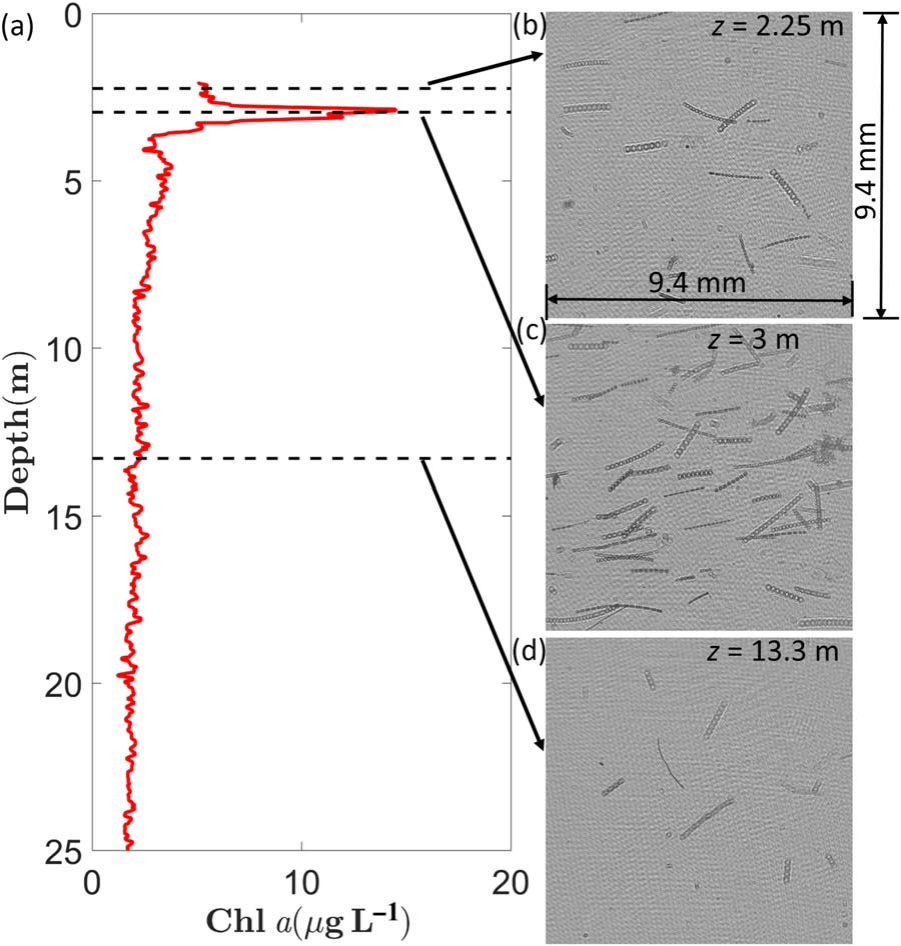
Sample data from Sta. 46, illustrating the presence of a “thin layer” of colonial diatoms. (**a**) Chl *a* profile for Sta. 46. Select raw holograms at depths marked with dashed lines: (**b**) above the layer at 2.25 m; (**c**) within the layer at 3 m; and (**d**) below the thin layer at 13.3 m.

Figure 3 shows the CTD density ( $\sigma_{T}$) profiles along with the chlorophyll concentration (Chl *a*) profiles as a function of depth in the water column, for all the stations. Chl *a* was derived using the absorption line‐height method described in Nardelli and Twardowski (2016). All depth values in this paper have been matched to the center of the FOV of the low resolution hologram sample area, unless otherwise noted. Station 39 and 40 were characterized by the lack of a strong pycnocline (Fig. 3a,b). There was a small peak in Chl *a* at ∼2.5 m in Sta. 39, while a broader peak was observed between ∼3.5 m and 5 m in Sta. 40. Below, there was a steady monotonic decrease through the rest of the water column. In contrast, Sta. 46 and 48 were characterized by the presence of a strong pycnocline between 2.7 m and 4 m (marked by dashed lines in figure). At both these stations, strong gradients in Chl *a* were observed, coincident with the upper region of the pycnocline (Fig. 3c,d). High Chl *a* within a small depth range are characteristic of “thin layers”—temporally coherent patches of highly concentrated phytoplankton that are widespread in coastal waters, and strongly correlated with the physical structure of the water column, e.g., usually the presence of pycnoclines (Dekshenieks et al. 2001; Sullivan et al. 2010; Durham and Stocker 2012). In Fig. 4, along with the corresponding Chl *a* profile for Sta. 48 (Fig. 4a), three sample holograms obtained above the thin layer (2.25 m, Fig. 4b), within the layer (3 m, Fig. 4c), and well below the layer (13.3 m, Fig. 4d) are shown. A clear difference in particle abundance can be observed between the holograms within and outside the thin layer (quantitative analysis follows in later sections).

Water column stability is characterized by the nondimensional Richardson number, which measures the relative importance of the stratification (buoyancy) to the mean shear:

(1) $$Ri=N^{2}/S^{2}$$

Here, N is the Brunt‐Vaisala (buoyancy) frequency, given by $N^{2}=-\left(g/\rho_{0}\right)\partial \rho /\partial z$, where *g* is the acceleration due to gravity, $\rho_{0}$ is the reference density, ρ is the local density, and *z* is the vertical coordinate; S=∂u/∂z represents the shear, i.e., the vertical gradient of the horizontal velocity. Simultaneous measurements of the velocity and density at exactly the same location and time were not possible; however, a reasonable estimate of *Ri* was obtained using the ADV shear and CTD density measurements, albeit spatially separated by a few cm. *Ri* varied from 4 to 15, easily exceeding the criterion necessary for stable stratification of the water column, i.e., *Ri* > 0.25 (Mann and Lazier 1991; Kundu and Cohen 2004).

### Flow structure

Background small‐scale shear and turbulence play an important role in determining the particle/plankton distribution and orientation in the water column. For example, several recent numerical and experimental studies have shown that turbulence can cause aggregation/clustering in both motile phytoplankton and zooplankton, leading to patchiness or heterogeneity in distribution patterns (Durham et al. 2013; De Lillo et al. 2014; Ardeshiri et al. 2016). Thus, the characterization of the flow field is critical to this analysis. Here, turbulence is quantified by calculating the TKE dissipation rate, *ε*, from the velocity field recorded by the ADV. While several approaches could be used to compute TKE dissipation estimates in spatially resolved oceanic flows (Nayak et al. 2015), the use of an ADV (single point sensor) necessitated estimating dissipation by fitting a line with a −5/3 slope to the inertial subrange of the velocity frequency spectrum and using Taylor's “frozen turbulence” hypothesis.

**Figure 5:**
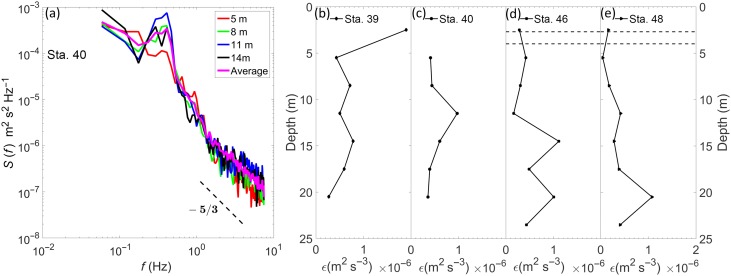
(**a**) Sample energy spectra calculated from the vertical velocity component using the ADV data at four different depth bins with mean spectrum overlaid, for Sta. 40. Profiles of TKE dissipation rate calculated from a −5/3 slope fit to the spectra obtained from the ADV data, sampled at 3 m depth bins, for (**b**) Sta. 39; (**c**) Sta. 40; (**d**) Sta. 46; and (**e**) Sta. 48.

From Kolmogorov's classical description of turbulence (Kolmogorov 1941; Pope 2000), the one‐dimensional energy spectrum in the inertial subrange is given by

(2) $$S_{ii}(\kappa_{i})=C\varepsilon^{2/3}\kappa_{i}^{-5/3}$$

where *i* = 1, 2, or 3, represents the velocity component in a particular direction, $\kappa_{i}$ is the wavenumber (rad m^−1^) in said direction, and *C* is a constant, value of which varies based on *i* (0.49 or 0.65, for the horizontal and vertical components, respectively). Where spatial data is lacking, one could use the Taylor's “frozen turbulence” hypothesis, to convert the temporal frequency spectra to the spatial domain, by relating wavenumber to frequency, as $\kappa_{3}=2\pi f/U$, where *f* is frequency, and *U* is mean velocity, which varied between 3 cm s^−1^ and 6 cm s^−1^ between stations. Using this, the dissipation rate is then provided by

(3) $$\varepsilon ={\left[\frac{S(f)f^{5/3}}{C}\right]}^{3/2}{\left(\frac{2\pi }{U}\right)}^{5/2}$$

where S(f) is the vertical velocity frequency spectrum. At least 256 instantaneous sampling points were required to generate a suitable spectrum from which dissipation can be estimated. The velocity data was divided into 3 m depth bins to achieve this minimum sampling criteria for each bin. Sample frequency spectra for Sta. 40 at 4 depth bins, along with the depth averaged spectrum overlaid are shown in Fig. 5a. In all the presented cases, the spectra exhibited a −5/3 slope at the higher frequencies, indicating that dissipation estimates from a fit to this inertial subrange were reasonable. Profiles of dissipation rate are presented in Fig. 5b–e. Dissipation at the first depth bin in Sta. 40 (2 m) is missing, as the spectra were noisy due to relatively shorter data length and did not present a −5/3 slope over which a reasonable fit could be made. The general trend in all profiles was that the turbulence levels were very low (∼10^−7^m^2^ s^−3^), with only four points among all stations exceeding 10^−6^ m^2^ s^−3^, with the highest values occurring at 2 m in Sta. 39. From the dissipation rate, one can obtain the Kolmogorov length scale representing the smallest eddies in the turbulent flow, as $\eta =\left(\nu^{3}/\varepsilon \right){}^{1/4}$. For the range of ε values at the different stations, the corresponding η values vary between 0.8 mm and 1.5 mm. The Batchelor scale which represents the length scale where diffusion and turbulence balance, is often used in the context of scalar transport in the ocean, e.g., for oxygen and nutrients (Batchelor 1959). Using the equation, $\lambda_{B}={\left(\frac{\nu D^{2}}{\varepsilon }\right)}^{1/4}$, where *D* = 10^−9^ m^2^ s^−1^ is the diffusion coefficient, $\lambda_{B}$ ranged from ∼25 μm to 45 μm between different stations and depths, at the lower end of the range typical in the ocean (Stocker 2012).

### Particle statistics

At Sta. 39, 46, and 48, holograms were acquired during both upcast and downcast profiles of the instrumentation package. At Sta. 40, the HOLOCAM acquired data only during the downcast. Most particle statistics discussed in the subsequent sections are based on the analysis of holograms acquired during downcast only. Holograms acquired during the upcast are used only in the context of discussing particle orientations and comparisons with PSDs in later sections. Table 1 provides a brief description of the number of holograms acquired, along with the particle counts during each of the station profiles.

**Table 1:** Hologram and particle counts at the analyzed sampling stations.

	Number of holograms (downcast)	Particle count (downcast)	Number of holograms (upcast)	Particle count (upcast)
Sta. 39	6600	152,105	1900	66,923
Sta. 40	8750	181,158	‐	‐
Sta. 46	8550	214,110	2250	91,112
Sta. 48	4400	117,275	2500	109,872

**Figure 6:**
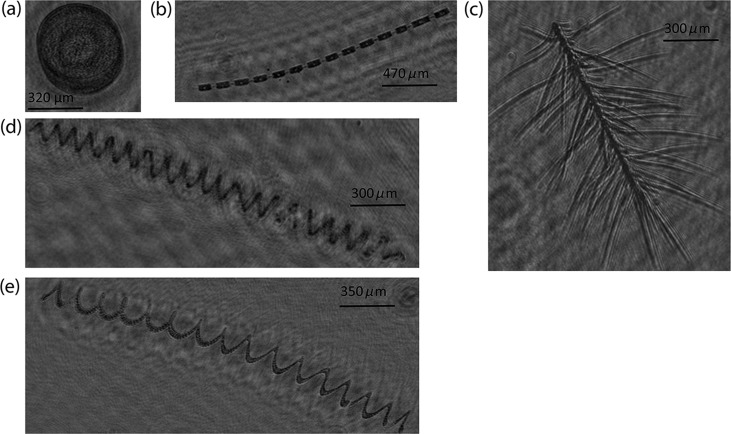
Sample species recorded in the holograms, representative of some of the particles included in the analysis. (**a**) *Coscinodiscus* sp.; (**b**) *D. brightwellii*; (**c**) *C*. cf. *concavicornis*; (**d**) *C. debilis*; and (**e**) *E. zodiacus*. The best in‐focus plane for each particle is chosen in its entirety, without using sub‐windows and creating a 2D composite image, as done for the particle analysis.

In total, 34,950 holograms were processed for this analysis, 28,400 during downcast, and 6650 during upcast. Particle statistics were calculated using every third hologram in a particular profile for the downcast, and every second hologram for the upcast, to avoid repeat counting of particles. Based on the descent rate and the hologram sampling frequency (15 Hz), we can see that between two successive holograms, the system displacement was ∼3.3–4 mm. Thus, between every third hologram, the system would have moved ∼9.9–12 mm, exceeding the hologram FOV (9.4 × 9.4 mm). Several hundred consecutive holograms from different portions in each of the four datasets were manually scanned to ensure that the sub‐sampling routine employed here prevents duplicate counting of the same particle in the automated processing steps. On the upcast, one in two holograms were used, as the ascent rate was at least double the descent rate. Each downcast contained at least 100,000 particles, totaling more than half a million particles over the course of the analysis, creating a robust database (Table 1). Sample in‐focus images of the most commonly observed particles (phytoplankton) during the profiles are shown in Fig. 6. The larger particle population throughout the water column was dominated by relatively few species of phytoplankton, particularly chain‐forming diatoms. At all stations, *Ditylum brightwellii* populated the entire water column, and dominated the thin layers in Sta. 46 and 48. Other commonly observed high aspect ratio diatom chains, included species from the genus *Chaetoceros*, e.g., *Chaetoceros* cf. *concavicornis* and *Chaetoceros debilis*, as well as *Eucampia zodiacus*. Species identifications were confirmed through independent analysis of water samples using a shipboard microscope.

### Particle concentration profiles

**Figure 7:**
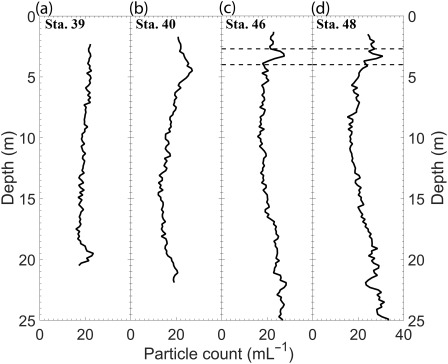
Particle concentration profiles (mL^−1^) using 20 cm depth bins for (**a**) Sta. 39; (**b**) Sta. 40; (**c**) Sta. 46; and (**d**) Sta. 48. Small concentration peaks are seen in Sta. 46 and 48 within the “thin layer.”

**Figure 8:**
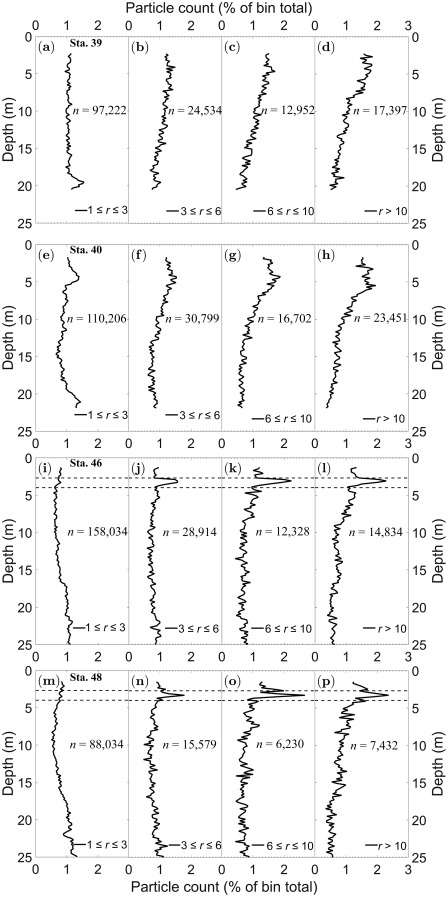
Particle concentration profiles (mL^−1^) for the four datasets, as a function of four aspect ratio bins, 1<r≤3, 3<r≤6, 6<r≤10, and r>10, respectively. (**a–d**) Sta. 39; (**e–h**) Sta. 40; (**i–l**) Sta. 46; and (**m–p**) Sta. 48.

Particle concentrations as a function of depth in the water column for all stations are presented in Fig. 7. Data presented include all particles within the resolved size ranges mentioned earlier, and have been depth‐binned at 20 cm intervals, i.e., each data point in the bin is given by $P_{v}=P/nV$ where $P_{v}$ is the particle concentration per mL, *P* is the number of particles in each hologram, *n* is the number of holograms in the respective depth bin, and *V* is the sample volume of each hologram. Trends differed among the various sampling stations. For Sta. 39, $P_{v}$ was steady with depth, ranging from ∼20 mL^−1^ to 25 mL^−1^ through the water column, with a slight peak at the lowest depths (Fig. 7a). The profile for Sta. 40 indicated a mild, broad peak in $P_{v}$ at ∼4.5 m, with a slow decrease with depth to 18 m. For Sta. 46 and 48, small peaks in $P_{v}$ were observed at depths corresponding to the thin layer (marked with dashed lines in Fig. 7c,d), where marked increases in Chl *a* were observed (Fig. 3). While this trend (slight increase in $P_{v}$ when Chl *a* peak is much stronger) might seem puzzling at first, it can be explained by examining how $P_{v}$ trends vary with the particle aspect ratio, *r*. Here, *r* for each particle is defined as ratio of the longest axis to the shortest axis. Figure 8 shows $P_{v}$ as a function of aspect ratio with depth for all the stations. Particles are grouped into four bins: 1<r≤3 consisting of smaller particles, 3<r≤6 mainly representative of the smallest diatom chains and other phytoplankton, and 6<r≤10 and r>10, both mostly comprised of the medium and long diatom chains, respectively (Fig. 8a–p). Particle count is expressed as a percentage of particles within a particular aspect ratio range in the depth bin over the entire number of particles of the same aspect ratio. For both Sta. 46 and 48, within the thin layer, the percentage of particles increased as aspect ratio increased (Fig. 8i–p). The absolute particle numbers (given in each figure) showed that high aspect ratio particles formed only a small fraction of the total particle count. While Chl *a* peaks were driven by the increased presence of these elongate chains, the actual total particle count did not increase by a significant percentage. Another interesting feature was the variation in particle concentration with depth: though most apparent in the profiles for Sta. 39 and 40 (Fig. 8a–d), broadly speaking, the gradient in $P_{v}$ increased as *r* increased, i.e., a proportionally higher percentage of longer chains occurred at the shallowest depths of the water column.

### PSDs

**Figure 9:**
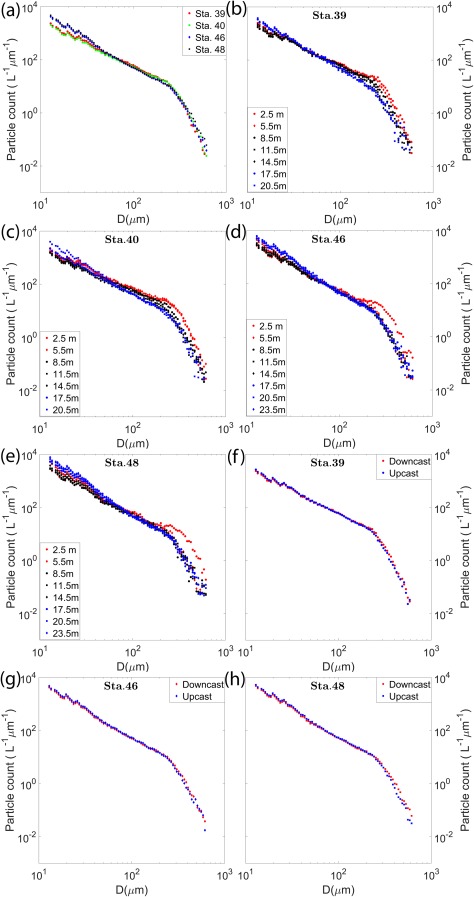
PSDs, where *D* is the particle equivalent spherical diameter, for (**a**) depth averaged data for all the four stations. For each individual station, PSD variations with depth are plotted using 3 m depth bins for (**b**) Sta. 39; (**c**) Sta. 40; (**d**) Sta. 46; and (**e**) Sta. 48. Comparisons between depth‐averaged PSDs for downcasts and upcasts at (**f**) Sta. 39; (**g**) Sta. 46; and (**h**) Sta. 48. Upcast data was not recorded at Sta. 40.

While the main focus of this article is particle orientation, a brief discussion of the PSDs at the different stations sheds light on the nature of the particle fields at the current location. It is important to note that in this PSD analysis, particle sizes are represented by the equivalent spherical diameter, *D*, which is measured as $\sqrt{4A/\pi }\;$, where *A* is the measured “filled” area of the particle. *A* is measured purely as a pixel count of the segmented image. However, in some cases, especially for larger particles, segmentation might result in losing certain pixels within the enclosed boundary of what constitutes the particle. In this case, we also include all the missing pixels to get a “filled” area, before using this information to obtain other parameters, e.g., equivalent spherical diameter. A popular instrument used in PSD studies is the Sequoia LISST‐100X, which provides size distributions of particles in the range 1.25–250 μm for the Type B model (Agrawal and Pottsmith 2000; Karp‐Boss et al. 2007; Buonassissi and Dierssen 2010). Although the low magnification HOLOCAM data cannot be used to reliably resolve particles near the lower size limit of the LISST‐100X, particle sizes nearly 40 times greater than the LISST‐100X's upper size limit can be fully characterized. While numerous approaches to model PSDs exist in literature (e.g., log‐normal, Gaussian, and gamma distributions), the most frequently used is the power‐law model, or so‐called Junge distribution, because of its ability to reasonably approximate natural PSD shapes with a single exponent parameter (Kitchen et al. 1982; Twardowski et al. 2001; Jonasz and Fournier 2007), which can be mathematically represented as

(4) $$n(D)=n_{0}{\left(D/D_{0}\right)}^{-\gamma }$$

where, $D_{0}$ is the reference diameter, $n_{0}$ is the differential particle concentration at $D_{0}$ (L^−1^ μm^−1^), and –*γ* is the slope of the Junge‐type distribution. Steeper slopes indicate that the particle population is dominated by smaller particles, while flatter slopes imply that large particles are more dominant. In our data, the particle sizes were grouped using logarithmically spaced size bins over the range of *D* from 11.6 μm to 1000 μm, and nondimensionalized by the width of each bin. Figure 9a shows the depth averaged PSDs during the downcasts for all stations. A clear knee was observed in the distributions around 250 μm, where the slopes on either side were quite different. Between 50 μm and 250 μm, the slopes ranged from 1.7 to 1.9 among the stations, whereas above 250 μm the slope was much steeper, in the range of 5.7–6.1. Below 50 μm, the PSDs were noisier, and deviated between runs such that Sta. 46 and 48 had a greater number of particles within this size range compared to Sta. 39 and 40. This is consistent with the trends seen in the particle count profiles in Fig. 7. In general, most studies focus on PSDs for what is assumed to be the optically relevant size range, i.e., < 100 μm, although a recent modeling study has shown that the contribution of larger particles (up to 2000 μm), might not be negligible in IOP studies (Davies et al. 2014) . In such cases, values of −*γ* range from 3 to 4 for marine particles (Kitchen et al. 1982; Buonassissi and Dierssen 2010; Reynolds et al. 2010). Slopes in our distributions were much lower, over part of similar size ranges, although a different size range applied here, and others have also found deviations from these values for specific cases (Jonasz and Fournier 2007; Buonassissi and Dierssen 2010). Furthermore, a brief review of existing literature by Reynolds et al. (2010) also showed that the Junge slope is sensitive to the particle size ranges over which the distribution is determined (Kitchen et al. 1982; Loisel et al. 2006).

To study variations in PSDs with depth within the same station, the same analysis was carried out after dividing the data into 3 m bins. Smaller depth bin sizes had inadequate particle counts. For Sta. 39 and 40 (Fig. 9b,c), while the two linear ranges in slopes existed at all depths, there was a clear steepening of the slope with depth between particle size ranges 50–250 μm. The slopes are indeed expected to be lower/flatter at the top, as we have already seen that the larger particles/chains dominate the upper layers of the water column. Trends were different in Sta. 46 and 48 (Fig. 9d,e). Below 5.5 m, the PSDs collapsed on top of each other with no significant differences. However, within the thin layer, there was a pronounced broad peak in particle concentration, centered on *D* = 300 μm, indicative of the significant increase in the presence of *Ditylum* chains within the layer, consistent with the raw hologram data presented in Fig. 4. In the size range below 50 μm, in all cases, slopes steepened with increasing depths, indicating enhanced presence of smaller particles (likely detrital matter) at the lowest depths. Furthermore, in the three cases where upcasts were recorded (Sta. 39, 46, and 48), the PSDs showed higher number of smaller sized particles (< 200 μm) when compared to the corresponding downcasts (Fig. 9f–h). While the difference is not very obvious due to the log scale of the plot, at the smallest bins, the difference in particle counts between the upcast and downcast was ∼300–400 L^−1^ μm^−1^. However, the trend reversed near the knee of the distribution, with slightly enhanced number of larger particles present in the downcast. It is likely that longer chains were broken due to the vigorous mixing that takes place during upcasts resulting in higher number of smaller particles. A second possibility is that this was simply a function of the randomized orientation leading to chains with major axes being aligned out of plane, causing the two‐dimensional (2D) projections to be significantly shorter (this aspect is explained further in the following section). Unfortunately, with the current data, we cannot say with any degree of certainty that either of these is the sole cause; indeed it is likely that both play an equally important role in the result. What this does highlight is the importance of sampling undisturbed particles in situ to ensure accurate PSD analysis, without using instrumentation which could fragment the particles during the quantification process.

The presence of multiple segmented linear slopes in PSDs has often been seen in the context of marine particles (Risović 1993; Jonasz and Fournier 2007). For particles in our size range, between *D* = 11.6–100 μm, shear coagulation supposedly dominates aggregation, and for larger particles, gravitational settling and differential sedimentation are the primary drivers, with steeper slopes occurring for the higher size ranges (Sullivan et al. 2005). Interestingly, a closely related study of bubble size distributions under breaking waves shows very similar linear regions with a distinct knee separating them (Deane and Stokes 2002), which the authors attribute to bubble breakage due to turbulence.

### Particle orientation analysis

Once major and minor axes of a particle were determined, the particle orientation angle was defined as the angle between the major axis of the particle and the horizontal axis of the hologram plane. The angles ranged from −90° to +90°, with 0° for a particle aligned in the horizontal plane and ± 90° for a particle oriented vertically. The angle was defined as positive if the particle had a positive slope (considering the bottom and left edges of the hologram as X and Y axes in a Cartesian coordinate system), and the angle was negative if the slope was negative. The orientation angle thus measured could be biased by the inclination of the profiling package as it descended through the water column. Therefore, the pitch readings from the ADV's in‐built tilt sensor were subtracted to adjust the orientation values accordingly before analysis. During profiles, the pitch of the system typically ranged between 6° ± 2°.

**Figure 10:**
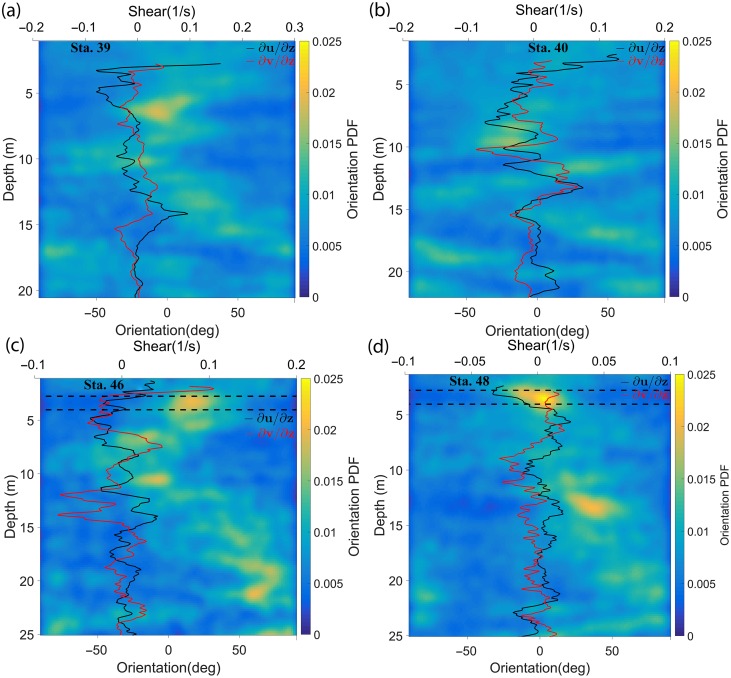
PDFs of orientation during downcasts with the velocity shear obtained from the ADV data overlaid for (**a**) Sta. 39; (**b**) Sta. 40; (**c**) Sta. 46; and (**d**) Sta. 48. 0° and ± 90° imply that the particle is oriented parallel to the horizontal or vertical axis of the HOLOCAM imaging plane, respectively (after instrument inclination has been accounted for).

Figure 10 shows the probability density functions (PDFs) of particle orientation over the entire water column, for downcasts at each station. Only particles with aspect ratio r > 3 were considered for this particular analysis to ensure accurate characterization of orientation PDFs. For Sta. 46 and 48, the locations of the pycnocline (and consequently the thin layer) have been marked with dashed lines (Fig. 10c,d). The shear strain rates, given by the vertical gradients of the in‐plane and out‐of‐plane horizontal velocity, ∂u/∂z and ∂v/∂z, obtained from the ADV data are overlaid on the PDF contours to facilitate comparisons between particle orientation and the local flow structure. While we do not resolve the particle orientations in the out‐of‐plane (axial) direction from the holograms to compare with ∂v/∂z, it is helpful to compare the relative magnitudes of ∂u/∂z and ∂v/∂z through the water column. ∂u/∂z and ∂v/∂z exhibited different trends between stations and depths. Peak values of ∂u/∂z occurred at the surface, at < 2.5 m at Sta. 39 and 40, dominating the corresponding ∂v/∂z values. At all other depths and stations, ∂u/∂z< 0.1 s^−1^. ∂v/∂z dominated only for depths < 2 m in Sta. 40, and in all other cases was < 0.1 s^−1^.

A few interesting trends stand out in the plots. First, in all four cases, peaks in the orientation PDFs were observed with varying intensities and at different depths. This observation showed that orientation was not perfectly random, as is often assumed. In Sta. 39, the PDF indicated preferential orientation centered around 0°, at 6 m depth (Fig. 10a), whereas at Sta. 40, the orientation PDF peaks were weaker, centered around −20° to the horizontal, between 8.5 m and 9.5 m (Fig. 10b). The strongest signatures however occurred within the thin layers at Sta. 46 and 48 (Fig. 10c,d, where broad peaks were centered at ∼20° and 0°, respectively. Secondary peaks outside the thin layer ranges centered at approximately −10° and −20° were also observed in Sta. 46, while the same phenomenon occurred at 35°, at ∼13 m in Sta. 48. Second, all the above‐mentioned PDF peaks were present at regions of very low in‐plane shear ( ∂u/∂z < 0.03 s^−1^). Third, while all regions of low shear did not necessarily contain particles exhibiting preferential orientation, near random orientation was the norm in regions of high in‐plane shear areas, e.g., ∂u/∂z > 0.1 s^−1^, within the top 3 m of Sta. 39 and 40. Comparing the dissipation profiles presented in Fig. 5 to the orientation PDFs for each corresponding station (Fig. 9), trends observed with shear applied to the dissipation as well, i.e., in all cases, preferential orientation was seen in regions of low dissipation (∼3 × 10^−7^ m^2^ s^−3^). While all regions of low dissipation did not exhibit preferential particle orientation, none of the high dissipation regions were associated with preferential orientation (e.g., at 2 m in Sta. 39).

**Figure 11:**
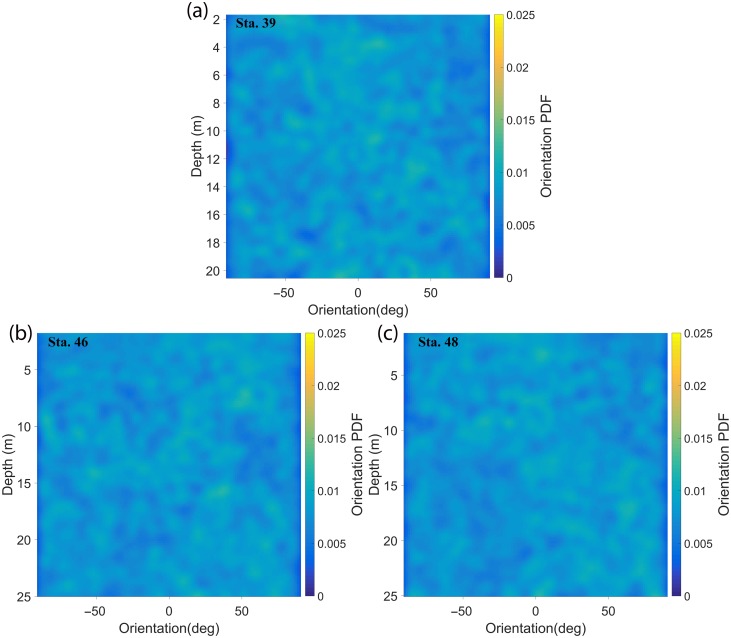
PDFs of orientation during upcasts for (**a**) Sta. 39; (**b**) Sta. 46; and (**c**) Sta. 48. The upcast on Sta. 40 was not recorded. 0° and ± 90° imply that the particle is oriented parallel to the horizontal or vertical axis of the HOLOCAM imaging plane, respectively (after instrument inclination has been accounted for).

A few other points should be noted in the context of this discussion. First, in the contour map for Sta. 46, the PDFs showed that particles were oriented ∼70° to the horizontal at 21 m and 25 m, respectively. This deviation from the norm, i.e., apparent preferential orientation to the vertical could be explained by re‐visiting the discussion on particle counts (Fig. 8). We could infer the total number of particles at a particular depth by summing the particle count over all aspect ratios > 3 (Fig. 8j–l). This indicated that there were barely 30 particles along 20 cm bins at either depth. Thus, even if one or two particles were randomly oriented, the low particle counts provide an unreasonably high PDF value of orientation. Another possible scenario is that these particles could actually be positioned such that their major axis lies predominantly out of plane, thus making the 2D projection seen in the x‐y plane appear vertical (explained in detail later in this section). Second, orientation PDFs as a function of depth for the upcasts during Sta. 39, 46, and 48 are presented in Fig. 11 (no upcast data was recorded for Sta. 40). As the HOLOCAM sample volume lies in the wake of the system, the flow was truly well‐mixed. So, one would expect the particle distribution and orientation to be random, as Fig. 11 indeed shows. These results further boost our belief that the preferential orientation observed during downcasts is not a manifestation of our profiling method. Another point to note is if the package was causing particles to be oriented in a particular manner within the sample volume, one would expect a similar effect at all depths, i.e., preferential alignment would be seen through the water column, irrespective of the flow conditions. Finally, it should be noted that all particle characteristics such as lengths and orientations in our analysis are based on a 2D projection of particles suspended in a 3D volume. For a line oriented in 3D space at a polar angle $0<\theta_{p}<\pi$, and an azimuthal angle $0<\theta_{a}<\pi$, the 2D projected angle *θ* is given by

(5) $$\theta =\tan^{-1}\left(\cot\theta_{p}/\cos\theta_{a}\right)$$

If we assume a uniform distribution of both $\theta_{p}$ and $\theta_{a}$, the orientation PDF showed increasing values at higher projected angles (*see* Fig. 16, Talapatra et al. 2013). For example, a diatom chain which is nearly horizontal, but predominantly aligned out of plane, will appear to be much shorter than its true length and vertically aligned in the projected plane. While this can indeed induce bias in the statistics, the images would be affected by projecting horizontally oriented particles as vertical. Thus, 2D projected angles under‐represent the true number of horizontally aligned particles/chains in our data. PDFs at lower angles thus represent minimum values, so that preferential orientation is likely more pronounced than our results depict.

It is important to recall that the Kolmogorov length scale *η*, varied between 0.8 mm and 1.5 mm among the different stations. Most, but not all, of the phytoplankton (and other particles) reside below this length scale, and thus experience a laminar shear. In his seminal work, Jeffery (1922) theorized that a rigid, ellipsoidal particle exposed to a laminar shear strain field, undergoes periodic “flipping” motions in a fixed orbit, with an angular velocity described by

(6) $$\omega =\dot{\theta }=\frac{S}{r^{2}+1}\left(r^{2}\sin^{2}\theta +\cos^{2}\theta \right)$$

where, θ is the instantaneous angle of orientation, *S* is the constant mean shear, and *r* is the aspect ratio of the particle. The time period for the particle to undergo one complete rotation is then

(7) $$T=\frac{2\pi }{S}(r+\frac{1}{r})$$

From Eqs. (6), (7), it can be seen that as *r* of a spheroid increases (assuming constant shear), ω decreases, with particles spending longer times aligned to the mean flow direction, before undergoing rapid flipping motions. The time taken to complete one rotation increases with increasing *r*. The following assumptions are inherent in the above mathematical formulation: (1) particles are large enough so that the Peclet number (ratio of advective to diffusive forces), Pe≫1, i.e., Brownian rotational diffusion is negligible; (2) particle inertia and particle‐particle interactions are negligible; and (3) Reynolds number based on particle size, $Re_{b}\ll 1$, so that viscous forces dominate (Gavze et al. 2012).

Although these assumptions represent a rather simplified model, it predicts the behavior of particles reasonably well in a wide range of experimental and numerical studies. For example, Trevelyan and Mason (1951) showed experimentally that within a rheological suspension undergoing shear in a Couette flow, cylindrical particles undergo periodic motions following Jeffery's theory. Further evidence that the theory holds good for particles even for nonlinear, wall‐bounded shear flows came from the numerical simulations of Ingber and Mondy (1994). Bretherton (1962) proved that orbits of a rotating particle of any shape, could be satisfactorily described by Jeffery's equations, provided the particle is modeled as an equivalent spheroid. A similar recent numerical study in the context of oceanic particles found the motion of diatoms with cellular projections (e.g., *Thalassiosira* sp.) could be accurately predicted using Jeffery's equations, provided an equivalent spheroid inscribes the cell, inclusive of the projections (Nguyen et al. 2011). The two laboratory studies most pertinent to this discussion on oceanic particles are by Karp‐Boss and Jumars (1998) and Marcos et al. (2011). The former studied the behavior of two chain‐forming diatom species exposed to a linear shear field. Results showed that *r* was strongly correlated to *T*, as predicted by Jeffery. However, values of *T* were consistently lower than those predicted by the theory, implying that for chains, Jeffery's equations overestimated the rotation periods (Karp‐Boss and Jumars 1998). Using a combination of laboratory experiments and numerical modeling, Marcos et al. (2011) showed that microbial particles do tend to preferentially orient to the flow direction under shear, with significant changes to the optical backscattering signature. With the general trend being that Jeffery's theory provides a good approximation in the laboratory under even complex particulate and flow conditions, we now have a basis to compare and frame our findings in an appropriate manner.

**Figure 12:**
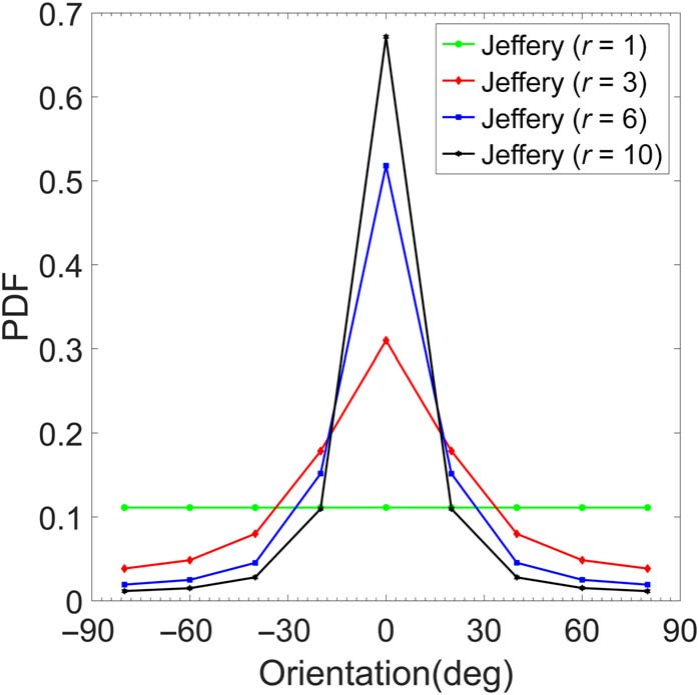
Modeled PDF values of orientation using Jeffery's equations for a spheroid in a simple shear flow for different aspect ratios. 0° indicates particle is oriented to the horizontal flow direction, and ± 90° indicates the particle is oriented perpendicular to the flow direction.

**Figure 13:**
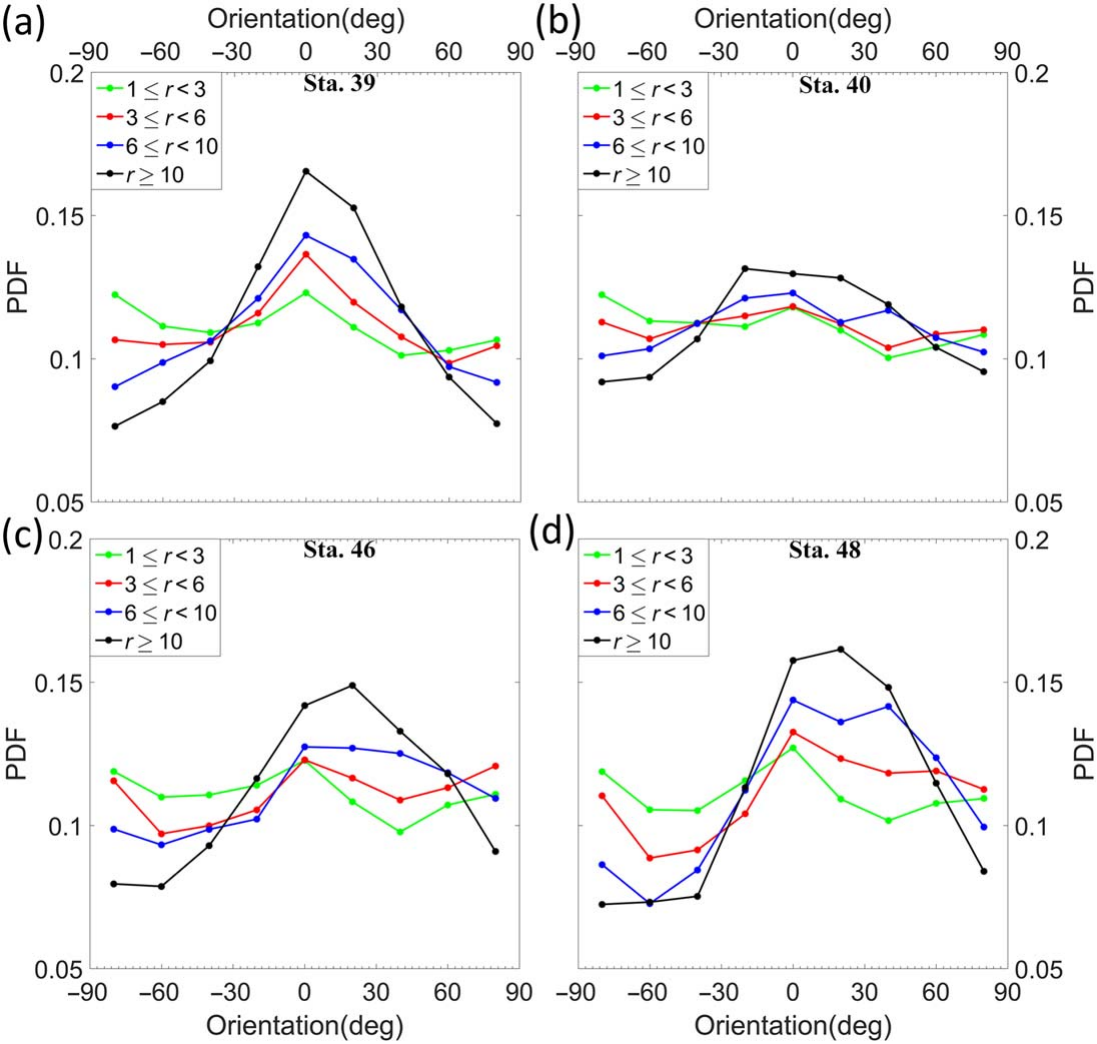
Orientation PDFs as a function of different aspect ratios for (**a**) Sta. 39; (**b**) Sta. 40; (**c**) Sta. 46; and (**d**) Sta. 48. 0° and ± 90° imply that the particle is oriented parallel to the horizontal or vertical axis of the HOLOCAM imaging plane, respectively (after instrument inclination has been accounted for).

Choosing a representative *S* = 0.1 s^−1^ and a certain number of time steps (10,000 in this case), the instantaneous orientation distribution over the entire rotation period, and hence the orientation PDF can be obtained. Initial particle orientation is assumed horizontal. The orientation angles from −90° to +90°, are segregated into nine 20° bins before calculating the PDFs. The modeled orientation PDFs for four different aspect ratios (*r* = 1, 3, 6, and 10), are shown in Fig. 12. PDF values around horizontal orientation angles are strongly positively correlated with *r*, as expected. Figure 13 shows the comparison of the measured orientation PDFs at all depths for each station as a function of *r*, using the same bins as in Fig. 7, i.e., 1<r≤3, 3<r≤6, 6<r≤10, and r>10. The orientation angles are binned as before in 20° bins. For all stations, a clear trend emerges: as *r* increases, the PDF peaks at nearly horizontal angles (Fig. 13a–d). While the actual PDF values are much lower than the modeled ones, the general shape of both PDFs is the same. Furthermore, naturally occurring particles are of different complex shapes and sizes, whereas the model is based on the assumption that the particles are perfect spheroids. The agreement between the modeled and observed PDFs is quite remarkable when placed in this context. This trend implies that longer chains (or higher aspect ratio particles) spend more time aligned to the horizontal direction, broadly agreeing with Jeffery's theory and the modeled PDFs. It is interesting to note that while changing shear values would change *T*, the time spent at each orientation relative to the total period is independent of shear, i.e., the orientation PDFs are purely a function of the aspect ratio.

## Summary and conclusions

A unique instrumentation suite was deployed in a field study at East Sound, Washington, to obtain simultaneous distributions of particle fields, shear and turbulence measurements, and optical data including absorption. A specific goal of this research was to examine whether particles in the undisturbed water column exhibited preferential orientation under different shear flow conditions. Four datasets containing particle statistics from holograms obtained during depth profiles under different mean shear and turbulence conditions form the basis of the discussion. The particles were composed chiefly of different phytoplankton including colonial diatom chains, marine snow, and detrital matter (near the bottom), with major axis lengths ranging from ∼14 μm to 11.6 mm (maximum measurable length in current configuration is 13.3 mm, the diagonal of the FOV), and areas ranging from 105 μm^2^ to 0.7 mm^2^, encompassing the lowest measurable limit to the area of the largest sampled particle. In two of these cases, a thin layer dominated by colonial diatom chains of the species *D. brightwellii*, was present ∼2.7–4 m below the surface, coincident with a strong pycnocline.

In all four datasets (as well as several others not discussed here), strong preferential particle orientation in the horizontal flow direction was observed, at various depths within the water column. On examining simultaneously obtained velocity shear profiles, it was found that orientation occurred in regions of low to near zero shear. Conversely, regions of high shear showed essentially randomly oriented particles. Similarly, higher turbulence levels correlated with random particle orientation (as one would expect), and all preferential orientation occurred in regions of low dissipation. Furthermore, PDF distributions of particle orientation showed that with increasing aspect ratios, particles spent more time oriented to the horizontal, agreeing remarkably well with Jeffery's theoretical model predictions for spheroids suspended in a simple shear flow. Our results clearly indicate nonrandom particle orientation in oceanic environments does occur, and seems to be nearly ubiquitous, at least for the observed shear and dissipation levels, both of which are wholly representative of the coastal and open oceans (Thorpe 2005) with the exception of boundary layer interactions and episodic, energetic events (Agrawal et al. 1992; Nayak et al. 2015). To the best of our knowledge, while briefly touched upon in two other studies (Malkiel et al. 1999; Talapatra et al. 2013), this phenomenon has not been studied extensively to date, and is often overlooked. Placed in this context, our results can prove to be significant as this phenomenon can have several consequences of note to phytoplankton ecology, ocean optics, and acoustics. For example, preferential alignment can change the ambient optical field by modifying scattering cross‐sections (Marcos et al. 2011), and recently published results on radiative transfer modeling for different diatom chain orientations clearly indicate that particles perpendicular to a light source receive more light than randomly oriented ones (Sun et al. 2016). This suggests morphologies favoring large aspect ratios, e.g., forming chains of cells, could be an ecological strategy to enhance light harvesting in oceanic phytoplankton. While the interaction between plankton morphology and light capture has received scarce attention, our current observations suggest there may exist complex interactions between morphology, fluid flow, and light capture. Since land plants have evolved the ability to actively orient to the sun's direction to maximize light absorption (phototropism) (Whippo and Hangarter 2006), evolution of strategies/morphologies in aquatic phytoplankton to enhance light capture through orientation should not be surprising. A detailed analysis of the optical effects is the focus of continuing work.

## Conflict of Interest

None declared.
